# Multidimensional characteristics of body composition in older adults and their relationship to common chronic diseases

**DOI:** 10.3389/fpubh.2026.1905131

**Published:** 2026-09-24

**Authors:** Mengfan Wei, Meng Li, Zhiquan Yang

**Affiliations:** Department of Rehabilitation Medicine, Beijing Zhongguancun Hospital/Zhongguancun Hospital, Chinese Academy of Sciences, Beijing, China

**Keywords:** association, body composition, chronic diseases, diabetes, dyslipidemia, older adults, hypertension

## Abstract

**Objective:**

This study aims to explore the multidimensional characteristics of body composition in adults aged 65 and older, analyze the association between body composition indicators and common chronic diseases such as hypertension, dyslipidemia, and diabetes, and provide reference for rehabilitation interventions for chronic diseases in the older adults.

**Methods:**

A total of 324 older adults individuals who underwent community health screenings were enrolled as study participants. An InBody 770 body composition analyzer was used to measure body composition indicators such as skeletal muscle mass (SMM), percent body fat (PBF), visceral fat level (VFL), and percent trunk fat (PTF). Data on chronic diseases were collected, and statistical analysis was performed using SPSS 26.0.

**Results:**

The prevalence of hypertension, dyslipidemia and diabetes among the study participants was 62.3, 53.7 and 19.8%, respectively. Significant gender and age differences were observed in the body composition of older adults: women had higher PBF and lower SMM, while adults aged 75 years and above had lower SMM and higher PBF compared with those aged 65–74 years. Regression analysis showed that higher PBF and higher VFL were independently associated with higher odds of hypertension; lower SMM and higher PBF were associated with dyslipidemia; and higher PBF, lower SMM and higher PTF were independently associated with higher odds of diabetes. Stratified analysis indicated that these associations exhibited heterogeneity by gender and age: the associations of excessive body fat with chronic diseases were more prominent in women, while the associations of low SMM with dyslipidemia and diabetes were stronger in older age groups.

**Conclusion:**

There are significant age and gender differences in body composition among the older adults population. Elevated body fat, visceral fat accumulation and insufficient SMM are closely associated with common chronic diseases in the older adults, and these associations present population-specific variations. The findings can provide reference evidence for the precise health management and rehabilitation intervention of chronic diseases in the older adults.

## 1 Introduction

With the rapid acceleration of population aging in China, the population aged 60 years and older had exceeded 297 million by the end of 2023, accounting for 21.1% of the total population, indicating that China has entered a stage of deep aging (1). Chronic diseases, as the primary threat to the health of the older adults, are showing a steadily rising prevalence. Surveys indicate that China currently has over 300 million people living with chronic diseases, including 270 million with hypertension (a prevalence rate as high as 25.2%), more than 100 million people with dyslipidemia (prevalence exceeding 10%), and 92.4 million people with diabetes (prevalence as high as 9.7%). Furthermore, these three conditions often coexist in pairs or all three together, significantly increasing the risk of complications such as cardiovascular events, stroke, and diabetic nephropathy, and placing a heavy burden on the healthcare system (2, 3). Body composition, as a core indicator reflecting nutritional status, metabolic function, and tissue composition, is closely linked to the onset, progression, and prognosis of chronic diseases. It has thus become a critical assessment target in geriatric health management (4). Traditional body composition assessment relies primarily on body mass index (BMI). However, BMI only reflects the ratio of body weight to height, and cannot distinguish tissue components such as fat, muscle and bone, making it difficult to accurately identify conditions such as masked obesity and sarcopenia (5). The InBody series of body composition analyzers, based on bioelectrical impedance analysis (BIA), can measure multidimensional indicators including skeletal muscle mass (SMM), percent body fat (PBF), visceral fat level (VFL) and body water distribution, providing a more comprehensive picture of the complex body composition characteristics of the older adults (6).

Although the association between body composition and metabolic chronic diseases has been widely explored, two key research gaps remain. First, most existing evidence is derived from middle-aged populations or clinical patient cohorts, and high-quality data focusing on community-dwelling adults aged 65 years and older in China are still relatively scarce. Older adults present unique physiological changes including age-related sarcopenia, visceral fat redistribution and altered body water homeostasis, which may lead to different association patterns between body composition and chronic diseases compared with younger populations; thus, existing conclusions cannot be directly extrapolated to the older adults group. Second, most studies only adopt single indicators such as BMI or total body fat percentage for analysis, and few systematically compare the associations of multi-dimensional body composition indices (overall fat, regional fat distribution, muscle mass) with multiple common metabolic chronic diseases simultaneously. This is insufficient to accurately capture the metabolic risks of masked obesity and sarcopenia in the older adults, which are characterized by normal BMI but abnormal internal tissue composition. Accordingly, this study enrolled 324 community-dwelling older adults aged 65 years and above. We used the InBody 770 body composition analyzer to measure multidimensional body composition indicators, and combined them with chronic disease diagnosis data from health examinations. This study aims to analyze age- and gender-related differences in body composition among the older adults population, clarify the associations and heterogeneous characteristics between body composition indicators and hypertension, dyslipidemia, and diabetes, so as to provide a scientific basis for developing individualized and precise rehabilitation intervention plans for chronic diseases in the older adults, and enrich evidence-based medical evidence for geriatric health management.

## 2 Research subjects and methods

### 2.1 Research subjects

This study was conducted from June 2023 to March 2024. Participants were community-dwelling older adults aged 65 years and older who participated in the annual free health examination at Beijing Zhongguancun Hospital. Consecutive sampling was adopted, and all eligible individuals who met the inclusion criteria during the recruitment period were invited to participate. Inclusion criteria: (1) Age ≥65 years, regardless of gender; (2) Voluntary participation in the study and signing of written informed consent. Exclusion criteria: (1) Severe organic diseases, including severe hepatic or renal insufficiency, malignant tumors, acute infections, or autoimmune diseases; (2) Limb amputation or severe motor dysfunction (e.g., hemiplegia) that would interfere with body composition testing; (3) Cognitive impairment that prevents completion of questionnaires and examinations; (4) Missing data rate ≥10%, or presence of extreme body composition outliers (BMI > 40 kg/m^2^ or PBF > 50%). The exclusion of extreme body composition values was implemented as a pre-specified quality control measure. On one hand, the accuracy of bioelectrical impedance analysis decreases significantly in individuals with morbid obesity due to altered body water distribution; on the other hand, such extreme values may introduce high-leverage bias in regression models and distort the estimation of association strengths. This practice is consistent with standard quality control protocols in population-based body composition studies. A total of 348 eligible older adults were invited to participate, and 324 completed all examinations and questionnaires with complete valid data, yielding an effective inclusion rate of 93.1%. This study was approved by the Ethics Committee of Beijing Zhongguancun Hospital. All participants were fully informed of the study purpose and procedures prior to enrollment, and signed written informed consent voluntarily.

### 2.2 Research methods

#### 2.2.1 Body composition assessment

Body composition measurements were performed using an InBody 770 body composition analyzer (Biospace, South Korea). All procedures were conducted by professionally trained medical staff in strict accordance with the instrument’s standard operating protocols: (1) Pre-test preparation: Participants were required to fast for 8–12 h and avoid strenuous exercise, alcohol, coffee and strong tea prior to testing. They wore light cotton clothing, removed footwear, and took off metal accessories such as watches and necklaces. (2) Testing procedure: Participants stood barefoot on the instrument’s electrode platform, held the electrode handles with both hands, maintained an upright posture with relaxed muscles, and avoided contact with surrounding metal objects. The device automatically completed data collection and analysis. (3) Measurement parameters: Indicators included baseline parameters (height, weight, BMI), fat-related parameters (PBF, VFL, visceral fat area [VFA], percent trunk fat [PTF]), muscle-related parameters (SMM, fat-free mass [FFM]), and water-related parameters (percent body water [PBW], intracellular water [ICW], extracellular water [ECW]).

#### 2.2.2 Diagnosis of chronic diseases

Diagnostic criteria were established in accordance with the “Chinese Guidelines for the Management of Hypertension in the Older adults 2023”, the “Chinese Guidelines for the Prevention and Treatment of Dyslipidemia in adults”, and the “Chinese Guidelines for the Prevention and Treatment of Type 2 Diabetes (2020 Edition)”. Hypertension: Systolic blood pressure ≥140 mmHg and/or diastolic blood pressure ≥90 mmHg, or a confirmed history of hypertension with current regular use of antihypertensive medications.· Dyslipidemia: Total cholesterol ≥5.2 mmol/L, triglycerides ≥1.7 mmol/L, low-density lipoprotein cholesterol (LDL-C) ≥ 3.4 mmol/L, high-density lipoprotein cholesterol (HDL-C) < 1.04 mmol/L (men) or <1.3 mmol/L (women), or a confirmed history of dyslipidemia with current regular use of lipid-lowering medications. Diabetes: Fasting blood glucose ≥7.0 mmol/L, 2-h postprandial blood glucose ≥11.1 mmol/L, glycated hemoglobin (HbA1c) ≥ 6.5%, or a previous confirmed diagnosis of diabetes with current regular use of antidiabetic medications or insulin therapy.

#### 2.2.3 General data collection

Relevant information was collected via a structured questionnaire and physical examination records: (1) Demographic characteristics: gender, age (divided into two groups: 65–74 years and ≥75 years). (2) Lifestyle factors: Smoking history (smoking defined as ≥1 year; otherwise, classified as non-smoking); alcohol consumption history (regular drinking was defined as alcohol intake ≥1 time per week; otherwise classified as non-drinking); physical activity (regular exercise was defined as exercise ≥3 times per week with ≥30 min per session; otherwise classified as irregular exercise). (3) Other: Past medical history, medication history, etc.

### 2.3 Statistical analysis

All statistical analyses were performed using SPSS 26.0. Continuous variables are presented as mean ± standard deviation, and categorical variables as frequencies (*n*) and percentages (%). Independent-samples *t*-test was used for between-group comparisons of continuous variables with two groups, and one-way analysis of variance (ANOVA) for multiple groups. The chi-square (*χ^2^*) test was applied for comparisons of categorical variables. Pearson correlation analysis was performed to assess linear associations between body composition indices and continuous metabolic biochemical markers. Multivariate logistic regression models were constructed to explore the associations between body composition indices and chronic diseases. Variables with *p* < 0.1 in univariate analysis were included in the multivariate models. The variance inflation factor (VIF) was calculated to evaluate multicollinearity among body composition indicators, with a VIF value <5 defined as no severe multicollinearity. All multivariate models were adjusted for age, gender, smoking history, alcohol consumption, regular physical exercise, and medication history to minimize confounding bias. The standardized physical examination panel only covered fasting glucose, lipid profiles and HbA1c for metabolic disease diagnosis. Hematological parameters (hemoglobin, hematocrit), iron metabolism biomarkers (ferritin) and inflammatory indicators (C-reactive protein [CRP], neutrophil-to-lymphocyte ratio [NLR]) were not available for the entire cohort, and thus could not be incorporated into regression models for confounding adjustment or mechanistic analysis. A two-tailed *p* < 0.05 was considered statistically significant.

## 3 Results

### 3.1 Demographic characteristics of the study participants

A total of 348 individuals participated in this study. After line-by-line review of the raw data, we excluded participants aged younger than 65 years (*n* = 2), those with missing key body composition or disease indicators (*n* = 19), and those who met the study exclusion criteria (*n* = 3). Finally, 324 participants were included in the final analysis. Among the 324 older adult participants, 116 were male (35.8%) and 208 were female (64.2%). The mean age was 72.9 ± 5.5 years; 201 participants (62.0%) were aged 65–74 years, and 123 (38.0%) were aged 75 years and older. In terms of lifestyle factors, 44 participants (13.6%) reported a smoking history, 30 (9.3%) reported alcohol consumption, and 87 (26.8%) engaged in regular physical exercise. The prevalence of chronic diseases in the study population was as follows: hypertension was identified in 202 participants (62.3%), including 118 (58.7%) in the 65–74 age group and 84 (68.3%) in the ≥75 age group; dyslipidemia was present in 174 participants (53.7%), including 97 (48.3%) in the 65–74 age group and 77 (62.6%) in the ≥75 age group; diabetes was diagnosed in 64 participants (19.8%), including 29 (14.4%) in the 65–74 age group and 35 (28.5%) in the ≥75 age group. Overall, 76 participants (23.5%) had two chronic conditions, 21 (6.5%) had three chronic conditions, and 83 (25.6%) had none of the three chronic diseases. The prevalence of hypertension, dyslipidemia and diabetes in the ≥75-year-old group was significantly higher than that in the 65–74-year-old group (χ^2^ = 4.215, 7.352, 10.683; *p* = 0.040, 0.007, 0.001, respectively).

### 3.2 Multidimensional characteristics of body composition in the older adults population

Test results for core body composition indicators in the older adults population showed: BMI (24.6 ± 3.1) kg/m^2^, PBF (32.8 ± 5.2) %, SMM (22.5 ± 4.4) kg, FFM (42.3 ± 7.6) kg, VFL (8.8 ± 3.0), PTF (36.2 ± 5.1) %, PBW (73.8 ± 0.5) %, ICW/ECW (1.62 ± 0.10).

When stratified by gender, significant differences were observed in core body composition indicators between male and female older adults. Muscle-related and water-related indicators were significantly higher in men, while fat-related indicators were significantly higher in women. For water-related indicators, men had significantly higher total body water (TBW), ICW and ECW than women (all *p* < 0.001); however, no significant difference in PBW was found between gender (*p* = 0.055), suggesting that the proportion of body water relative to FFM remains relatively stable in the older adults population and is not markedly affected by gender. Detailed stratified results are presented in Table 1.

**Table 1:** Baseline characteristics of body composition indicators in the overall older adults population (mean ± SD).

Indicator	Total (*n* = 324)	Male (*n* = 116)	Female (*n* = 208)	*t*	*p*
BMI (kg/m^2^)	24.6 ± 3.1	24.9 ± 3.0	24.4 ± 3.1	1.386	0.167
PBF (%)	32.8 ± 5.2	28.2 ± 4.1	35.5 ± 4.7	−14.982	<0.001
SMM (kg)	22.5 ± 4.4	26.9 ± 3.4	19.9 ± 2.6	18.567	<0.001
FFM (kg)	42.3 ± 7.6	48.8 ± 6.3	38.7 ± 6.0	12.093	<0.001
VFL	8.8 ± 3.0	8.4 ± 2.8	9.0 ± 3.1	−1.793	0.074
PTF (%)	36.2 ± 5.1	32.3 ± 4.5	38.7 ± 5.0	−12.436	<0.001
PBW (%)	73.8 ± 0.5	73.9 ± 0.4	73.7 ± 0.5	1.924	0.055
ICW/ECW	1.62 ± 0.10	1.64 ± 0.09	1.61 ± 0.10	2.287	0.023

BMI, body mass index; PBF, percentage body fat; SMM, skeletal muscle mass; FFM, fat-free mass; VFL, visceral fat level; PTF, percentage trunk fat; PBW, percentage body water; ICW, intracellular water; ECW, extracellular water; SD, standard deviation.

When stratified by age group, the 65–74-year-old group had significantly higher SMM and FFM than the ≥75-year-old group, while PBF and PTF were significantly lower than those in the ≥75-year-old group. No significant differences in BMI, VFL and PBW were observed between the two age groups. Detailed results are presented in Table 2.

**Table 2:** Comparison of body composition characteristics between different age groups (mean ± SD).

Indicator	65–74 years group (*n* = 201)	≥75 years group (*n* = 123)	*t*	*p*
BMI (kg/m^2^)	24.5 ± 3.0	24.7 ± 3.2	−0.578	0.564
PBF (%)	32.1 ± 5.1	33.8 ± 5.2	−3.015	0.003
SMM (kg)	23.1 ± 4.3	21.6 ± 4.4	2.897	0.004
FFM (kg)	43.2 ± 7.4	40.9 ± 7.7	2.541	0.011
VFL	8.7 ± 2.9	8.9 ± 3.1	−0.592	0.554
PTF (%)	35.6 ± 5.0	37.2 ± 5.1	−2.678	0.008
PBW (%)	73.8 ± 0.5	73.7 ± 0.5	0.987	0.324
ICW/ECW	1.63 ± 0.10	1.61 ± 0.10	1.563	0.119

BMI, body mass index; PBF, percentage body fat; SMM, skeletal muscle mass; FFM, fat-free mass; VFL, visceral fat level; PTF, percentage trunk fat; PBW, percentage body water; ICW, intracellular water; ECW, extracellular water; SD, standard deviation.

### 3.3 Differences in body composition across different chronic disease conditions

*Hypertension*: Compared with the non-hypertension group, the hypertension group had significantly higher PBF, PTF and VFL, as well as significantly lower SMM and FFM. No significant differences in BMI or PBW were observed between the two groups (Table 3).

**Table 3:** Comparison of body composition between the hypertension and non-hypertension groups (mean ± SD).

Indicator	Hypertension group (*n* = 202)	Non-hypertensive group (*n* = 122)	*t*	*p*
BMI (kg/m^2^)	24.8 ± 3.2	24.3 ± 3.0	1.298	0.195
PBF (%)	34.0 ± 5.0	30.7 ± 5.1	5.643	<0.001
SMM (kg)	21.9 ± 4.2	23.6 ± 4.6	−3.217	0.001
FFM (kg)	41.5 ± 7.5	43.6 ± 7.6	−2.345	0.020
VFL	9.6 ± 2.9	7.5 ± 2.8	5.782	<0.001
PTF (%)	37.3 ± 4.9	34.4 ± 4.8	4.762	<0.001
PBW (%)	73.7 ± 0.5	73.9 ± 0.5	−1.243	0.215
ICW/ECW	1.61 ± 0.10	1.63 ± 0.10	−1.385	0.167

BMI, body mass index; PBF, percentage body fat; SMM, skeletal muscle mass; FFM, fat-free mass; VFL, visceral fat level; PTF, percentage trunk fat; PBW, percentage body water; ICW, intracellular water; ECW, extracellular water; SD, standard deviation.

*Dyslipidemia*: Compared with the non-dyslipidemia group, the dyslipidemia group had significantly higher BMI, PBF, PTF and VFL, along with significantly lower SMM and FFM. No significant difference in PBW was found between the two groups (Table 4).

**Table 4:** Comparison of body composition between the dyslipidemia and non-dyslipidemia groups (mean ± SD).

Indicator	Dyslipidemia group (*n* = 174)	Non-dyslipidemia group (*n* = 150)	*t*	*p*
BMI (kg/m^2^)	25.0 ± 3.2	24.0 ± 3.0	2.854	0.003
PBF (%)	34.1 ± 4.9	30.9 ± 5.2	5.718	<0.001
SMM (kg)	21.8 ± 4.3	23.7 ± 4.4	−3.896	<0.001
FFM (kg)	41.4 ± 7.4	43.5 ± 7.7	−2.587	0.010
VFL	9.5 ± 2.9	7.7 ± 3.0	5.109	<0.001
PTF (%)	37.4 ± 4.8	34.6 ± 4.9	4.683	<0.001
PBW (%)	73.7 ± 0.5	73.9 ± 0.5	−1.619	0.107
ICW/ECW	1.61 ± 0.10	1.63 ± 0.10	−1.592	0.112

BMI, body mass index; PBF, percentage body fat; SMM, skeletal muscle mass; FFM, fat-free mass; VFL, visceral fat level; PTF, percentage trunk fat; PBW, percentage body water; ICW, intracellular water; ECW, extracellular water; SD, standard deviation.

*Diabetes*: Compared with the non-diabetes group, the diabetes group had significantly higher PBF, PTF and VFL, and significantly lower SMM, FFM and PBW. No significant difference in BMI was observed between the two groups (Table 5).

**Table 5:** Comparison of body composition between the diabetic and non-diabetic groups (mean ± SD).

Indicator	Diabetes group (*n* = 64)	Non-diabetic group (*n* = 260)	*t*	*p*
BMI (kg/m^2^)	24.9 ± 3.3	24.5 ± 3.1	0.986	0.325
PBF (%)	35.7 ± 4.8	32.2 ± 5.1	4.973	<0.001
SMM (kg)	20.7 ± 4.1	22.8 ± 4.4	−3.762	<0.001
FFM (kg)	39.8 ± 7.2	42.7 ± 7.6	−2.684	0.008
VFL	10.2 ± 2.8	8.4 ± 3.0	4.159	<0.001
PTF (%)	38.9 ± 4.7	35.7 ± 5.0	4.526	<0.001
PBW (%)	73.5 ± 0.5	73.9 ± 0.5	−2.987	0.003
ICW/ECW	1.60 ± 0.11	1.63 ± 0.10	−1.893	0.059

BMI, body mass index; PBF, percentage body fat; SMM, skeletal muscle mass; FFM, fat-free mass; VFL, visceral fat level; PTF, percentage trunk fat; PBW, percentage body water; ICW, intracellular water; ECW, extracellular water; SD, standard deviation.

Pearson correlation analysis was further conducted to explore the linear associations between body composition indices and continuous metabolic biochemical markers (Supplementary Table S1). Briefly, PBF, VFL and PTF showed moderate positive correlations with HbA1c, total cholesterol and triglycerides (all *p* < 0.05). In contrast, SMM and FFM showed weak negative correlations with these metabolic biomarkers (all *p* < 0.05). BMI and PBW only presented partial weak correlations with blood lipid and glucose indicators. These linear correlation trends were consistent with the intergroup differences shown in Tables 3–5.

### 3.4 Analysis of the association between body composition measures and chronic diseases

Logistic regression analysis was performed to investigate the associations of body composition indicators with hypertension, dyslipidemia and diabetes. Variables with *p* < 0.1 in univariate analysis were entered into multivariate logistic regression models, which were adjusted for age, gender, smoking history, alcohol consumption, regular physical exercise and medication history to identify independent associations. Detailed results of the overall associations are presented in Table 6.

**Table 6:** Logistic regression analysis of body composition indices and chronic diseases (Univariate OR/Multivariate OR, 95% CI).

Indicator	Hypertension		Dyslipidemia		Diabetes	
	Univariate OR (95% CI)	Multivariate OR (95% CI)	Univariate OR (95% CI)	Multivariate OR (95% CI)	Univariate OR (95% CI)	Multivariate OR (95% CI)
PBF	1.095 (1.047–1.145)	1.076 (1.020–1.135)	1.078 (1.031–1.127)	1.061 (1.008–1.117)	1.115 (1.048–1.187)	1.084 (1.014–1.159)
PTF	1.075 (1.026–1.127)	1.054 (0.998–1.113)	1.069 (1.021–1.120)	1.047 (0.993–1.104)	1.089 (1.026–1.156)	1.062 (0.997–1.131)
SMM	0.942 (0.893–0.993)	0.966 (0.910–1.026)	0.883 (0.830–0.940)	0.902 (0.822–0.990)	0.859 (0.775–0.952)	0.889 (0.797–0.992)
VFL	1.362 (1.189–1.561)	1.297 (1.078–1.560)	1.293 (1.136–1.471)	1.138 (0.948–1.366)	1.426 (1.203–1.693)	1.183 (0.960–1.458)
FFM	0.968 (0.935–1.002)	0.980 (0.943–1.019)	0.957 (0.927–0.988)	0.969 (0.935–1.005)	0.948 (0.909–0.989)	0.962 (0.918–1.008)
BMI	1.061 (1.005–1.120)	1.021 (0.956–1.091)	1.086 (1.030–1.145)	1.034 (0.972–1.100)	1.058 (0.975–1.148)	1.015 (0.926–1.112)
PBW	0.912 (0.785–1.059)	0.947 (0.808–1.110)	0.905 (0.788–1.039)	0.939 (0.806–1.093)	0.652 (0.478–0.890)	0.751 (0.535–1.053)

PBF, percentage body fat; PTF, percentage trunk fat; SMM, skeletal muscle mass; VFL, visceral fat level; FFM, fat-free mass; BMI, body mass index; PBW, percentage body water; OR, odds ratio; CI, confidence interval. Multivariate models were adjusted for age, gender, smoking history, alcohol consumption, regular physical exercise, and medication history. All odds ratios correspond to a per 1-unit increase in each body composition indicator (% for PBF and PTF; kg for SMM, FFM and BMI; grade for VFL).

*Hypertension*: Univariate analysis showed that PBF, PTF, VFL, SMM and FFM were significantly associated with hypertension (*p* < 0.05), among which the first three indicators showed positive associations, while the latter two showed negative associations. After adjustment for age, gender, smoking history, alcohol consumption, regular physical exercise and medication history, multivariate logistic regression analysis revealed that higher PBF and higher VFL were independently associated with hypertension (both *p* < 0.05). Specifically, for every 1% increase in PBF, the odds of hypertension were 7.6% higher; for every 1-grade increase in VFL, the odds of hypertension were 29.7% higher. After full adjustment, the associations of the remaining indicators with hypertension were not statistically significant (*p* > 0.05).

*Dyslipidemia*: Univariate analysis showed that PBF, PTF, VFL, SMM, FFM and BMI were significantly associated with dyslipidemia (*p* < 0.05), with positive associations observed for PBF, PTF, VFL and BMI, and negative associations observed for SMM and FFM. Multivariate logistic regression analysis with adjustment for the above covariates and medication history indicated that lower SMM and higher PBF were independently associated with dyslipidemia (both *p* < 0.05). Specifically, for every 1 kg increase in SMM, the odds of dyslipidemia were 9.8% lower; for every 1% increase in PBF, the odds of dyslipidemia were 6.1% higher. After full adjustment, the associations of the remaining indicators with dyslipidemia were not statistically significant (*p* > 0.05).

*Diabetes*: Univariate analysis showed that all included body composition indicators (PBF, PTF, VFL, SMM, FFM, BMI and PBW) were significantly associated with diabetes (*p* < 0.05), with positive associations for PBF, PTF, VFL and BMI, and negative associations for SMM, FFM and PBW. Multivariate logistic regression analysis with full adjustment including medication history revealed that higher PBF and lower SMM were independently associated with diabetes (*p* < 0.05). Specifically, for every 1% increase in PBF, the odds of diabetes were 8.4% higher; for every 1 kg increase in SMM, the odds of diabetes were 11.1% lower. After full adjustment, the associations of the remaining indicators with diabetes were not statistically significant (*p* > 0.05).

### 3.5 Stratified analysis of the association between body composition indicators and chronic diseases

*Stratified by gender*: stratified analyses with adjustment for age, smoking history, alcohol consumption, regular physical exercise and medication history showed that the strength of the associations of higher PBF and higher VFL with hypertension was greater in females than in males. For dyslipidemia, the positive association of higher PBF was stronger in females, while the inverse association of higher SMM was more pronounced in males. For diabetes, the positive associations of higher PBF and higher PTF with increased disease odds were stronger in females than in males (Table 7).

**Table 7:** Analysis of the association between body composition indices and chronic diseases by gender (multivariate OR, 95% CI).

Indicator	Hypertension		Dyslipidemia		Diabetes	
	Male	Female	Male	Female	Male	Female
PBF	1.060 (0.990–1.135)	1.085 (1.013–1.162)	1.050 (0.990–1.114)	1.071 (1.004–1.142)	1.063 (0.972–1.162)	1.102 (1.014–1.197)
VFL	1.246 (1.004–1.547)	1.352 (1.072–1.705)	1.101 (0.896–1.352)	1.169 (0.944–1.448)	1.154 (0.899–1.482)	1.211 (0.946–1.550)
SMM	0.957 (0.875–1.047)	0.973 (0.900–1.052)	0.868 (0.760–0.991)	0.915 (0.820–1.021)	0.899 (0.773–1.045)	0.879 (0.768–1.006)
PTF	1.041 (0.973–1.114)	1.068 (1.003–1.137)	1.032 (0.962–1.107)	1.062 (0.994–1.135)	1.042 (0.958–1.134)	1.078 (1.000–1.162)
BMI	1.011 (0.931–1.098)	1.029 (0.947–1.118)	1.025 (0.941–1.117)	1.042 (0.954–1.138)	1.004 (0.893–1.129)	1.025 (0.922–1.140)

PBF, percentage body fat; VFL, visceral fat level; SMM, skeletal muscle mass; PTF, percentage trunk fat; BMI, body mass index; OR, odds ratio; CI, confidence interval. All stratified models were adjusted for age, smoking history, alcohol consumption, regular physical exercise, and medication history. All odds ratios correspond to a per 1-unit increase in each body composition indicator (% for PBF and PTF; kg for SMM and BMI; grade for VFL).

*Stratified by age*: compared with the 65–74-year-old group, the inverse association of higher SMM with dyslipidemia and diabetes, as well as the positive association of higher PTF with both conditions, were stronger in the ≥75-year-old group. No significant difference in the strength of the associations of PBF and VFL with hypertension was observed between the two age groups (Table 8).

**Table 8:** Analysis of the association between body composition indices and chronic diseases by age group (multivariate OR, 95% CI).

Indicator	Hypertension		Dyslipidemia		Diabetes	
	65–74 years	≥75 years	65–74 years	≥75 years	65–74 years	≥75 years
PBF	1.072 (1.005–1.143)	1.080 (1.004–1.162)	1.055 (0.991–1.123)	1.069 (1.000–1.143)	1.076 (0.978–1.184)	1.096 (1.005–1.195)
VFL	1.289 (1.048–1.586)	1.322 (1.035–1.688)	1.107 (0.898–1.365)	1.166 (0.915–1.486)	1.248 (0.955–1.630)	1.301 (1.019–1.662)
SMM	0.970 (0.897–1.049)	0.961 (0.876–1.054)	0.927 (0.829–1.037)	0.870 (0.763–0.992)	0.915 (0.789–1.061)	0.845 (0.731–0.977)
PTF	1.034 (0.967–1.106)	1.077 (1.002–1.158)	1.034 (0.964–1.109)	1.069 (0.995–1.149)	1.040 (0.955–1.133)	1.080 (0.998–1.169)
BMI	1.014 (0.934–1.101)	1.030 (0.940–1.129)	1.028 (0.946–1.118)	1.041 (0.949–1.142)	1.009 (0.903–1.128)	1.021 (0.907–1.150)

PBF, percentage body fat; VFL, visceral fat level; SMM, skeletal muscle mass; PTF, percentage trunk fat; BMI, body mass index; OR, odds ratio; CI, confidence interval. All stratified models were adjusted for smoking history, alcohol consumption, regular physical exercise, and medication history. All odds ratios correspond to a per 1-unit increase in each body composition indicator (% for PBF and PTF; kg for SMM and BMI; grade for VFL).

## 4 Discussion

### 4.1 Multidimensional characteristics and population differences in body composition among the older adults

The results of this study demonstrate that the body composition of the older adults population is characterized by the coexistence of elevated PBF and insufficient SMM, with significant age- and gender-related differences. The overall PBF of the study participants was 32.8 ± 5.2%, which is significantly higher than that of middle-aged and young adults in China (7). PBF was significantly higher in women (35.5 ± 4.7%) than in men (28.2 ± 4.1%), which is consistent with previous studies showing that although men have a higher prevalence of obesity, women tend to have a higher percentage of body fat (8). The potential mechanisms underlying age-related body fat accumulation have been widely explored in prior research. Age-related metabolic decline is considered a major contributor to increased PBF in older adults (9). With advancing age, reduced thyroid function and decreased insulin sensitivity may contribute to increased fat synthesis and impaired lipolysis. When combined with reduced daily physical activity and lower energy expenditure in older adults, these changes may further promote fat accumulation (10). In women, the significant decline in estrogen levels after menopause has been reported to drive a shift in fat distribution from the limbs to the trunk and visceral compartments (11). This may not only lead to a more pronounced increase in PBF but also a greater tendency toward visceral fat accumulation, which may partially explain the significantly higher trunk fat percentage observed in women compared with men in this study (12). In terms of skeletal muscle mass, the mean SMM of the study population was 22.5 ± 4.4 kg, with 26.9 ± 3.4 kg for men and 19.9 ± 2.6 kg for women, reflecting generally low muscle mass in this older adults cohort. Age-related SMM decline is a common physiological change, but the rate and magnitude of muscle loss are modulated by multiple factors including gender and physical activity habits. Men typically have a higher baseline SMM in early adulthood and a relatively slower rate of muscle loss with aging, which may account for their significantly higher muscle mass compared with women (13). This study also found that participants aged 75 years and older had significantly lower SMM and higher PBF than those aged 65–74 years, indicating that advancing age is closely associated with deterioration of body composition. Consistent with previous findings, the prevalence of sarcopenia and obesity tends to increase significantly with age in older adults (14).

In addition, the prevalence of overweight and obesity among older adults in this study was 52.7%, which is largely consistent with the 51.7% prevalence of overweight and obesity reported among older adults living in the relevant study community in Beijing (15). Notably, some participants with BMI within the normal range (18.5–23.9 kg/m^2^) had PBF above the clinically recommended thresholds (>25% for men, >30% for women), indicating the presence of masked obesity. This finding suggests that relying solely on BMI to evaluate body fat status in older adults has notable limitations. Multi-frequency BIA devices such as the InBody analyzer can accurately distinguish fat and muscle tissue and quantify key indicators including PBF and visceral fat level, enabling effective identification of masked obesity and providing more comprehensive reference for health risk assessment in the older adults (16).

### 4.2 Association and mechanisms between abnormal body composition and three common chronic diseases

#### 4.2.1 Hypertension

After full adjustment for confounding factors including medication history, multivariate logistic regression analysis showed that higher PBF and higher VFL were independently associated with increased odds of hypertension. Specifically, for every 1% increase in PBF, the odds of hypertension were 7.6% higher; for every 1-grade increase in VFL, the odds were 29.7% higher. These findings are generally consistent with results from numerous domestic and international studies (17, 18).

The potential mechanisms underlying the association between adipose accumulation and hypertension have been widely investigated in prior studies, and are mainly summarized as follows. First, inflammatory response derived from adipose tissue. Excess adipose tissue, especially visceral fat, has been recognized as an active inflammatory organ that may secrete abundant pro-inflammatory cytokines such as tumor necrosis factor-α (TNF-α) and interleukin-6 (IL-6) (19). These cytokines may impair vascular endothelial function, reduce the release of vasodilators including nitric oxide (NO), and enhance vasoconstrictive responses, which may consequently contribute to blood pressure elevation (20). In addition, inflammatory activation may stimulate the renin-angiotensin-aldosterone system (RAAS), promote water and sodium retention, increase circulating blood volume, and further aggravate blood pressure elevation (21). Second, mechanical compression and metabolic disturbance induced by visceral fat accumulation. Visceral fat deposition may directly compress intra-abdominal vessels, increase peripheral vascular resistance, and thus may contribute to elevated blood pressure (22). Meanwhile, the active metabolism of visceral fat generates large amounts of free fatty acids, which enter the liver via the portal venous system and may trigger insulin resistance. Insulin resistance may in turn increase renal sodium reabsorption, further driving blood pressure elevation (23). In addition, the significant intergroup difference in PTF and its positive association with hypertension in univariate analysis also reflect the influence of fat distribution on cardiovascular risk. Accumulating evidence has shown that trunk fat, especially abdominal fat, has a stronger association with cardiovascular diseases than peripheral limb fat (24), supporting the view that central obesity is closely related to elevated cardiovascular risk (25). Notably, all the above mechanistic explanations are inferred from published literature. Given the lack of systemic inflammatory biomarkers in our cohort, the mediating role of inflammatory pathways cannot be directly verified in this study.

#### 4.2.2 Dyslipidemia

The results of this study indicate that low SMM, high PBF, and high PTF were independently associated with dyslipidemia. Specifically, for every 1 kg increase in SMM, the odds of dyslipidemia were 9.8% lower; while a 1% increase in PBF was associated with 6.1% higher odds of dyslipidemia. These findings support the close association between “sarcopenia-adiposity syndrome” and metabolic disorders (26).

The potential mechanisms linking body composition changes to dyslipidemia have been elaborated in previous studies. As the body’s largest metabolic organ, reduced SMM may directly affect lipid metabolism (27). Skeletal muscle is the primary site for the oxidative breakdown of triglycerides; low SMM may lead to a decline in triglyceride oxidative metabolism, causing triglycerides to accumulate in the liver, blood vessels, and other sites, which may contribute to hypertriglyceridemia (28). Concurrently, reduced SMM may be associated with decreased insulin sensitivity; insulin resistance may further exacerbate lipid metabolism disorders, promote the synthesis of very-low-density lipoprotein (VLDL) cholesterol, lower high-density lipoprotein (HDL) cholesterol levels, and increase the likelihood of dyslipidemia (29). The association between high PBF and dyslipidemia may be explained by the following pathways: excessive adipose tissue may promote a decrease in lipoprotein lipase activity, leading to reduced triglyceride breakdown; simultaneously, inflammatory factors secreted by adipose tissue may impair hepatic lipid metabolism, increase cholesterol synthesis, and further exacerbate dyslipidemia (30). Furthermore, the independent association between trunk fat percentage and dyslipidemia suggests a closer link between central obesity and dyslipidemia, consistent with previous findings that abdominal fat accumulation is positively correlated with the odds of dyslipidemia (31). Notably, since hematological parameters such as hemoglobin and iron metabolism indicators were not collected in this study, we cannot rule out the potential confounding effect of chronic disease anemia on muscle mass loss and lipid metabolism disorders, and the underlying pathway needs to be further confirmed by studies with complete laboratory data.

#### 4.2.3 Diabetes

The results of this study indicate that high PBF, low SMM, and high PTF were independently associated with diabetes. Specifically, for every 1% increase in PBF, the odds of diabetes were 8.4% higher; for every 1 kg increase in SMM, the odds of diabetes were 11.1% lower, which is consistent with the conclusions of domestic and international studies (32, 33). The potential mechanism underlying the association between high PBF and diabetes is mainly related to insulin resistance, as reported in previous studies (34). Inflammatory factors secreted by excessive adipose tissue (especially visceral fat) may inhibit insulin receptor signaling pathways, reduce insulin sensitivity, and result in decreased glucose uptake and utilization, which may contribute to elevated blood glucose levels (35). Concurrently, free fatty acids produced by adipose tissue may accumulate within pancreatic β-cells, impairing β-cell function and reducing insulin secretion, thereby further exacerbating glucose metabolism disorders (36). The mechanism linking low SMM to diabetes is primarily related to glucose metabolism: skeletal muscle is the primary site of insulin-mediated glucose uptake, accounting for more than 70% of total body glucose uptake (37). Low SMM may lead to decreased glucose uptake capacity and slowed glucose clearance rate, which in turn may be associated with insulin resistance and diabetes (38). Notably, as noted in the limitations section, the potential confounding effect of chronic disease anemia cannot be ruled out due to missing hematological data.

### 4.3 Heterogeneity in the association between body composition and chronic diseases and limitations of the study

The stratified analysis results of this study showed that the associations of body composition with hypertension, dyslipidemia and diabetes presented significant age- and gender-specific heterogeneity, which has important implications for targeted health management in the older adults.

Regarding gender heterogeneity, the strength of the association between high PBF and high VFL and hypertension was stronger in women than in men. Similarly, the associations of higher PBF and higher PTF with diabetes were more prominent in women. In contrast, the association between low SMM and dyslipidemia was stronger in men. Potential explanations for these gender differences have been discussed in previous studies, mainly related to physiological structural differences and hormone level variations between gender. After entering old age, declining estrogen levels in women may exacerbate fat metabolism disorders and contribute to body fat accumulation, especially in the trunk region, which may be associated with higher odds of hypertension and diabetes (39). In men, the relatively faster rate of muscle loss with aging may lead to a more prominent association between low SMM and abnormal lipid metabolism, which may explain the stronger association with dyslipidemia observed in this study (40). This gender heterogeneity indicates that gender differences should be fully considered when exploring the associations between body composition and chronic diseases in the older adults.

Regarding age heterogeneity, the associations of low SMM and high PTF with dyslipidemia and diabetes were stronger in the ≥75-year-old group than in the 65-74-year-old group, while no significant age difference was observed in the associations of PBF and VFL with hypertension. This finding is consistent with the age-related trajectory of body composition in older adults. After the age of 75, the rate of muscle loss accelerates with a significant decline in SMM, and trunk fat accumulation further increases (41). These age-related body composition changes may lead to more pronounced disturbances in lipid and glucose metabolism, which may strengthen the observed associations with dyslipidemia and diabetes. For hypertension, as its occurrence is influenced by multiple factors, the role of body composition abnormalities did not show significant differences across age groups in this study. This age-specific heterogeneity further enriches the evidence on body composition and chronic diseases in the older adults, and clarifies the differences in the associations of body composition abnormalities with metabolic disorders across different age groups.

Several limitations of this study should be acknowledged. First, this study adopted a cross-sectional design with one-time measurements of body composition and disease status. Therefore, only statistical associations between body composition indicators and chronic diseases can be identified, and the causal direction cannot be determined. Second, all participants were recruited from a single community in Beijing with relatively homogeneous backgrounds. Thus, the generalizability of the findings to older adults across different regions and socioeconomic levels is limited. Third, hematological, iron metabolism and inflammatory biomarkers (hemoglobin, ferritin, CRP, NLR) were not available in this cohort. On one hand, we cannot disentangle whether low SMM is secondary to metabolic disorders or chronic anemia, nor can we verify the inflammatory mediating pathway between visceral fat accumulation and metabolic diseases; all relevant mechanistic discussions are inferred from published literature rather than confirmed by our own data. On the other hand, the absence of complete blood count and anemia status may lead to potential misestimation of skeletal muscle mass and sarcopenia status measured by bioelectrical impedance analysis, introducing systematic bias in the assessment of core exposure indicators. Fourth, although all multivariate models have been adjusted for available confounders including medication history, detailed information on drug type, dosage and duration, as well as quantitative dietary habits, exercise volume, disease duration and socioeconomic status, were not collected. Therefore, residual confounding bias may still exist. In addition, regular use of antihypertensive, antidiabetic and lipid-lowering drugs may affect the distribution of body fluid components; in particular, diuretic antihypertensive agents can alter intracellular and extracellular water balance, which may introduce certain measurement bias in BIA-derived body water and muscle mass indices and cannot be fully controlled in the current analysis. Finally, body composition was measured by bioelectrical impedance analysis (InBody 770). Its measurement accuracy for indicators such as SMM and PBF is inferior to the gold standard dual-energy X-ray absorptiometry (DXA), which may introduce certain measurement error. Nevertheless, this study systematically evaluated the associations of multiple body composition indicators with common metabolic chronic diseases in community-dwelling older adults, providing valuable reference evidence for chronic disease risk stratification and health management in the older adults population.

## 5 Conclusion

This study showed that there are significant gender and age differences in body composition among adults aged 65 years and older. Higher PBF, higher VFL and lower SMM are closely associated with three common chronic diseases: hypertension, dyslipidemia and diabetes. Furthermore, these associations present age- and gender-specific heterogeneity. The above body composition indicators can provide reference for chronic disease risk assessment in the older adults, and offer basic data support for further exploring the pathological mechanisms of chronic diseases and developing targeted health management strategies. In clinical practice, body composition testing may be incorporated into routine health examinations for the older adults, especially for those aged 75 years and older women. Key indicators such as PBF, SMM and VFL deserve close attention to identify individuals with abnormal body composition at an early stage. Meanwhile, community-based health management for the older adults should be strengthened, and health education on body composition should be promoted to improve the quality of life of the older adults population. Given the cross-sectional design of this study, the causal direction between body composition abnormalities and chronic diseases cannot be determined. Future large-sample prospective longitudinal studies are warranted to verify the predictive value of body composition indicators for the onset and progression of chronic metabolic diseases.

## Data Availability

The original contributions presented in the study are included in the article/Supplementary material, further inquiries can be directed to the corresponding author.

## Glossary

ANOVA: analysis of variance
BIA: bioelectrical impedance analysis
BMI: body mass index
CRP: C-reactive protein
DXA: dual-energy X-ray absorptiometry
ECW: extracellular water
FFM: fat-free mass
HbA1c: glycated hemoglobin
HDL-C: high-density lipoprotein cholesterol
ICW: intracellular water
IL-6: interleukin-6
LDL-C: low-density lipoprotein cholesterol
NLR: neutrophil-to-lymphocyte ratio
NO: nitric oxide
PBF: percent body fat
PBW: percent body water
PTF: percent trunk fat
RAAS: renin-angiotensin-aldosterone system
SMM: skeletal muscle mass
TBW: total body water
TNF-α: tumor necrosis factor-α
VFA: visceral fat area
VFL: visceral fat level
VIF: variance inflation factor
VLDL: very-low-density lipoprotein
OR: odds ratio
CI: confidence interval
SD: standard deviation
