# Supplementary material for: Multidimensional characteristics of body composition in older adults and their relationship to common chronic diseases
Source: Front Public Health. 2026 Sep 24;14:1905131. doi: 10.3389/fpubh.2026.1905131 (PMC13646107; doi:10.3389/fpubh.2026.1905131)
Supplement: Supplementary file 1 [file Table_1.docx]

**Supplementary Table S1** Pearson correlation coefficients between body composition indicators and continuous metabolic biochemical markers

| Body composition indicator | FPG (mmol/L) | | HbA1c (%) | | TC (mmol/L) | | TG (mmol/L) | | LDL-C (mmol/L) | | HDL-C (mmol/L) | |
| --- | --- | --- | --- | --- | --- | --- | --- | --- | --- | --- | --- | --- |
|  | r | *P* | r | *P* | r | *P* | r | *P* | r | *P* | r | *P* |
| BMI(kg/m²) | 0.21 | <0.05 | 0.22 | <0.05 | 0.25 | <0.001 | 0.29 | <0.001 | 0.24 | <0.001 | -0.2 | <0.05 |
| PBF (%) | 0.30 | <0.001 | 0.32 | <0.001 | 0.28 | <0.001 | 0.35 | <0.001 | 0.27 | <0.001 | -0.25 | <0.001 |
| PTF (%) | 0.26 | <0.001 | 0.27 | <0.001 | 0.24 | <0.001 | 0.31 | <0.001 | 0.23 | <0.001 | -0.22 | <0.001 |
| VFL | 0.29 | <0.001 | 0.30 | <0.001 | 0.26 | <0.001 | 0.33 | <0.001 | 0.25 | <0.001 | -0.24 | <0.001 |
| SMM (kg) | -0.20 | <0.05 | -0.21 | <0.05 | -0.18 | <0.05 | -0.24 | <0.001 | -0.17 | <0.05 | 0.19 | <0.05 |
| FFM (kg) | -0.18 | <0.05 | -0.19 | <0.05 | -0.16 | <0.05 | -0.21 | <0.001 | -0.15 | <0.05 | 0.17 | <0.05 |
| PBW (%) | -0.07 | >0.05 | -0.08 | >0.05 | -0.06 | >0.05 | -0.09 | >0.05 | -0.05 | >0.05 | 0.06 | >0.05 |
| ICW/ECW | -0.05 | >0.05 | -0.06 | >0.05 | -0.04 | >0.05 | -0.07 | >0.05 | -0.04 | >0.05 | 0.05 | >0.05 |

Abbreviations: FPG, fasting plasma glucose; HbA1c, glycated hemoglobin; TC, total cholesterol; TG, triglycerides; LDL-C, low-density lipoprotein cholesterol; HDL-C, high-density lipoprotein cholesterol; BMI, body mass index; PBF, percentage body fat; PTF, percentage trunk fat; VFL, visceral fat level; SMM, skeletal muscle mass; FFM, fat-free mass; PBW, percentage body water; ICW, intracellular water; ECW, extracellular water.

Note: Pearson correlation analysis was performed for all continuous variables in the total cohort (n=324). r represents the correlation coefficient; P<0.05 was considered statistically significant.
